# Study on the Effect of Emulsifiers on the Properties of Oleogels Based on Olive Oil Containing Lidocaine

**DOI:** 10.3390/ijms252011067

**Published:** 2024-10-15

**Authors:** Sonia Kudłacik-Kramarczyk, Alicja Przybyłowicz, Anna Drabczyk, Weronika Kieres, Robert P. Socha, Marcel Krzan

**Affiliations:** 1 Jerzy Haber Institute of Catalysis and Surface Chemistry, Polish Academy of Sciences, 8 Niezapominajek St., 30-239 Krakow, Poland; alicja.przybylowicz@student.pk.edu.pl (A.P.); weronikakieres1605@gmail.com (W.K.); robert.socha@cbrtp.pl (R.P.S.); marcel.krzan@ikifp.edu.pl (M.K.); 2 Faculty of Mechanical Engineering, Cracow University of Technology, 37 Jana Pawła II Av., 31-864 Krakow, Poland; 3 CBRTP SA—Research and Development Center of Technology for Industry, Ludwika Waryńskiego 3A St., 00-645 Warsaw, Poland; anna.drabczyk@cbrtp.pl

**Keywords:** oleogels, lidocaine, long-term stability, rheology, emulsions, physicochemical properties

## Abstract

Oleogels are semi-solid materials that consist primarily of liquid oil immobilized in a network of organized structural molecules, which provide stability and maintain the oil in the desired shape. Due to their structure, oleogels can stabilize large amounts of liquid, making them excellent carriers for active substances, both lipophilic and hydrophilic. This study presents the synthesis methodology and investigations of olive oil-based oleogels, which are among the healthiest and most valuable vegetable fats, rich in unsaturated fatty acids and antioxidants such as vitamin E. Two types of surfactants were used: TWEEN 80, which lowers surface tension and stabilizes emulsions, and SPAN 80, which acts in oil-dominated phases. The oleogels were enriched with lidocaine, an active substance commonly used as a pain reliever and local anesthetic. This research characterized the obtained oleogels regarding their medical applications, paying particular attention to the influence of surfactant type and amount as well as the active substance on their physicochemical properties. Structural analyses were also conducted using Fourier transform infrared (FTIR) spectroscopy, alongside rheological and sorption studies, and the wettability of the materials was evaluated. The stability of the obtained oleogels was verified using the MultiScan MS20 system, allowing for an assessment of their potential suitability for long-term pharmaceutical applications. The results indicated that SPAN-stabilized oleogels exhibited better stability and favorable mechanical properties, making them promising candidates for medical applications, particularly in pain relief formulations.

## 1. Introduction

Oleogels are semi-solid materials that consist mainly of liquid oil immobilized in a network of organized structural molecules that provide stability and hold the oil in the desired shape [1,2,3]. The properties of oleogels are due to their structure, which allows them to stabilize large amounts of liquid oil. These materials function as excellent carriers for lipophilic and hydrophilic active substances [4]. Due to their texture, they are easily adapted to a variety of applications, such as skin hydration and protection from external agents or controlled drug release [3,5]. Oleogels are also used extensively in the food industry, including, among others, as replacements for common fats in food. In addition, they enable the manufacture of healthier products without losing flavor and textural properties [6,7,8].

In this paper, the synthesis methodology and investigations on olive oil-based oleogels are presented. Olive oil is one of the healthiest and most valuable vegetable fats. It is rich in unsaturated fatty acids and contains antioxidants, including vitamin E. It also demonstrates anti-inflammatory properties [9,10,11]. In cosmetics, this oil is widely used as a moisturizer. Due to the presence of antioxidants, it helps fight skin aging [12,13].

In the synthesis, two types of surfactants, namely, TWEEN 80 and SPAN 80, were used. Span 80 (sorbitan monooleate) and Tween 80 (polysorbate 80) are widely used emulsifiers in oleogel formulations [14,15,16]. Span 80, an emulsifier with a low HLB (Hydrophilic–Lipophilic Balance) value of 4.3, favors the formation of w/o (water-in-oil) emulsions, while Tween 80, due to its high HLB value of 15.0, prefers to stabilize o/w (oil-in-water) emulsions [17,18]. The key parameter that determines their properties is HLB (Hydrophilic–Lipophilic Balance), which describes the balance between the hydrophilic and lipophilic properties of an emulsifier and allows an assessment of how much a given surfactant prefers to dissolve in water or oil. The HLB value determines an emulsifier’s ability to stabilize a particular type of emulsion—low HLB values indicate a more hydrophobic character, which favors the stabilization of w/o (water-in-oil) emulsions, while high HLB values indicate greater hydrophilic properties, which supports the formation of o/w (oil-in-water) emulsions. This makes the HLB parameter a key indicator for selecting the right emulsifier for specific applications and conditions, such as oil-in-oil or water-in-water phase stability in oleogels [19,20,21]. HLB values for hydrophilic surfactants typically range from 8 to 18, while those for hydrophobic surfactants are below [22,23,24]. This selection of emulsifiers allows for the precise control of the structure and rheological properties of oleogels by adjusting phase distribution and intermolecular interactions. In the present study, these two surfactants were selected to verify the impact of emulsifiers with different HLB values on selected physicochemical properties of oleogels including, among others, wettability, stability, and sorption ability. The use of surfactants with different HLB values makes it possible to characterize their interactions with the oil and aqueous phases, which is important for understanding the mechanisms shaping the structure and stability of the final oleogels.

Importantly, the oleogels were enriched with lidocaine. This active substance is widely used as an arrhythmic drug and a local anesthetic [25]. Its action is based on blocking sodium channels found in the membranes of nerve cells, resulting in rapid local anesthesia. It is prepared in the form of injections, sprays, gels, ointments, and patches [26,27].

In the literature, several investigations focused on the characteristics of systems containing both olive oil and lidocaine have been so far presented. For example, in the work of Dogrul et al. [28], olive oil was used as one of the oil phases in microemulsions containing lidocaine hydrochloride. It was observed that the use of olive oil as an oil phase led to an increase in the droplet size of microemulsions in which aqueous solutions of lidocaine hydrochloride were dispersed. The increase in the size of these aqueous droplets in the emulsion was due to the interaction of oleic acid present in the oil with lecithin and ethanol (used as surfactant and co-surfactant, respectively), which affected the structure and stability of the film at the water–oil interface. In addition, a reduction in the rate of drug release (only 12.6% of the drug was released within 6 h) was also observed for the systems obtained with olive oil compared to those obtained with Miglyol. In turn, the main goal of the research of Zhang et al. [29] was to develop microemulsions without co-emulsifiers to minimize toxicity and irritation associated with their use. To achieve this, olive oil was mixed with α-linolenic acid and linoleic acid, and vitamin E succinate was used as an auxiliary oil. The final optimal formulation contained 3.23% olive oil to improve the skin permeability of lidocaine and increase its retention. Key findings of the study demonstrated that the use of olive oil in combination with fatty acids and vitamin E significantly improved the skin permeation of lidocaine and prolonged the duration of anesthesia compared to a commercial cream. The microemulsion showed good stability, minimal irritation, and significant anesthetic efficacy. Next, the study of Daryab et al. [30] evaluated gel-based microemulsions as vehicles for the transdermal delivery of lidocaine, including the use of olive oil and various surfactants and co-emulsifiers. A key goal was to investigate how olive oil, in combination with other reagents, affects the solubility and permeability of lidocaine through the skin. The use of olive oil in microemulsions was shown to increase the solubility of lidocaine. In turn, studies performed by Qiao et al. [31] evaluated various formulations of microemulsions containing lidocaine, including those using olive oil as the oil phase. The main objective of the research was to optimize the microemulsions for better transdermal penetration and retention of lidocaine in the skin while reducing the amount of surfactants, which should reduce toxicity and the risk of skin irritation. The study used multi-object optimization algorithms to achieve the best pharmaceutical properties. The key findings of the study indicate that a microemulsion with olive oil (1.11%) formulated with 0.64% α-linolenic acid, 2.54% linoleic acid, 0.71% vitamin E succinate, 15% surfactant, 5% lidocaine, and 75% water provided the highest skin permeation of lidocaine (0.17 μg/cm^2^·s) and retention in the skin (0.74 mg/cm^2^). Another optimal formulation with a higher ratio of olive oil to fatty acids (1.42) and less surfactant content (13.6%) improved anesthesia duration, achieving a lidocaine retention of 0.80 mg/cm². These formulations showed better penetration and retention of lidocaine compared to other investigated systems.

However, no studies focusing on investigations of olive oil-based oleogels containing lidocaine and discussing the application of two types of surfactants in the synthesis of these oleogels have been presented so far. Hence, the aim of this research was to characterize the obtained oleogels in terms of their application for medical purposes, with particular attention paid to the influence of the surfactant type (and amount) and active substance used on their physicochemical properties. In the course of the conducted research, the structure of the oleogels was also characterized using FT-IR spectroscopy to verify potential interactions between the oleogel and the introduced active substance. Performed studies also included the analysis of rheological and sorption properties of oleogels, as well as their wettability. In addition, the stability of the obtained materials over time was verified using the MultiScan system to determine their potential suitability for long-term pharmaceutical applications.

## 2. Results

### 2.1. Stability of Obtained Oleogels within the Period of Two Months

**Figure 1:**
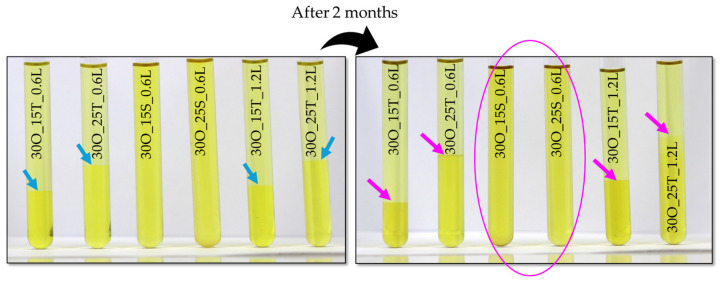
Images presenting the appearance of oleogel samples directly after synthesis (**left**) and after two months (**right**) (blue arrows demonstrate the level of oleogel in the test tubes after the synthesis; pink arrows demonstrate the level of oleogel in the test tubes after 2 months storage; pink circle shows samples in the case of which any difference has been observed).

**Figure 2:**
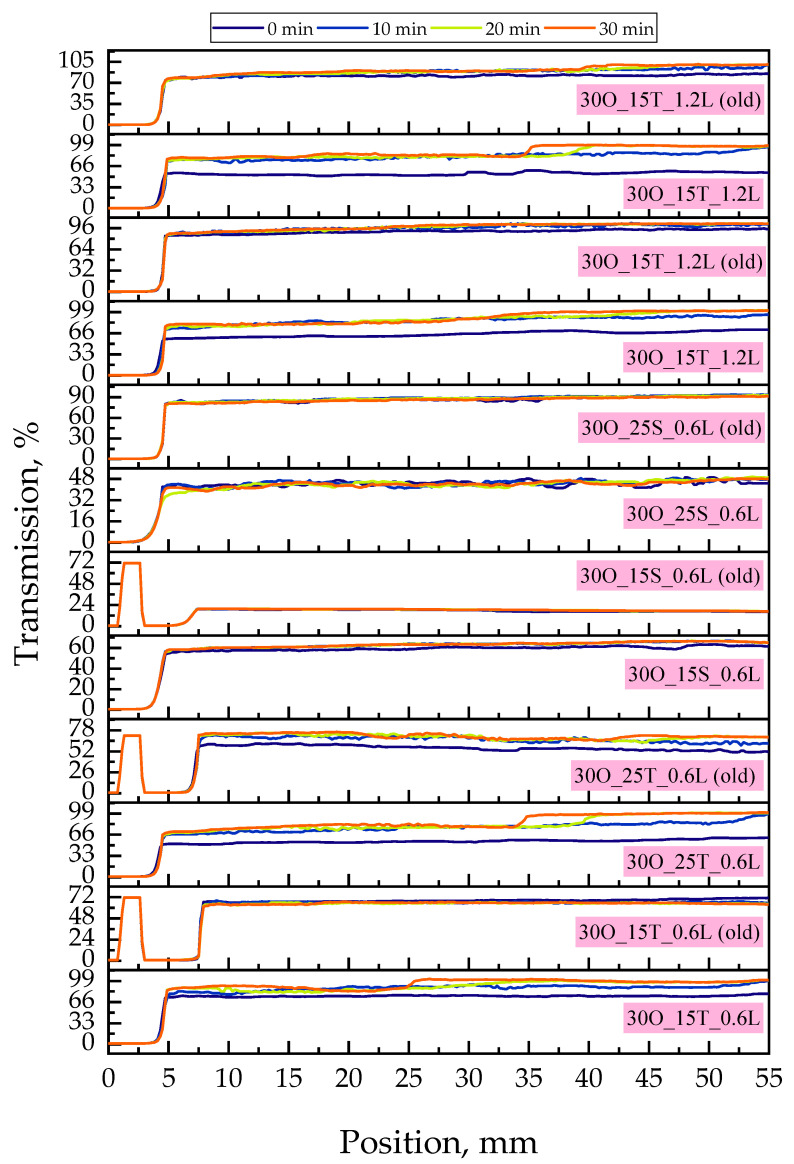
Results of the stability analysis via the MultiScan system for the oleogel samples; analysis after synthesis and after two months (old).

Figure 1 presents the appearance of the obtained samples directly after their preparation and after two months, while in Figure 2, the results of the conducted analysis of oleogels’ stability via the MultiScan system are compiled.

**Figure 3:**
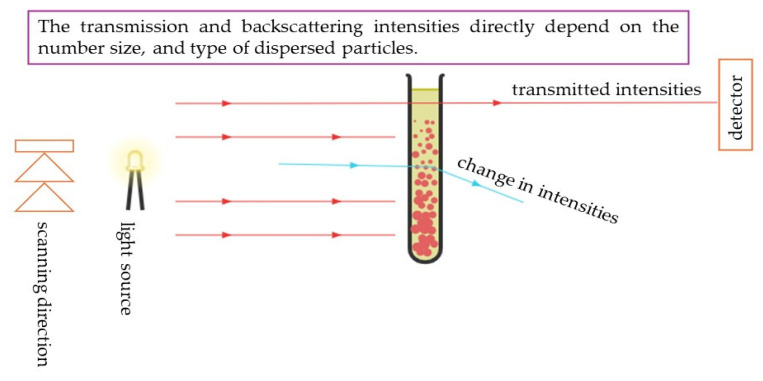
Schematic of the transmission measurement process using the MultiScan MS 20 device (The DataPhysics Instruments GmbH, Filderstadt, Germany) for the tested materials.

The principle of operation of this MultiScan system is illustrated in the diagram below (Figure 3).

The MultiScan system operates by analyzing the transmission and absorption of light through the oleogel samples. It utilizes a range of wavelengths to assess the structural integrity and stability of the emulsions over time. By comparing the transmission spectra of freshly synthesized samples with those stored for two months, the MultiScan system can identify changes in phase separation, which may indicate destabilization. Additionally, the device helps evaluate the interactions between the oleogels and the active substance, lidocaine, providing insights into how these factors influence the overall physicochemical properties of the materials. Overall, this method is crucial for assessing the long-term suitability of the oleogels for pharmaceutical applications.

### 2.2. Results of FT-IR ATR Spectroscopy

**Figure 4:**
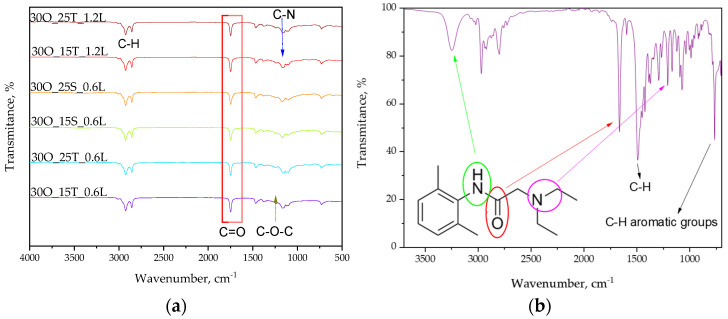
FT-IR spectra of oleogel samples (**a**) and lidocaine (**b**).

The results of spectroscopic analysis are shown below. Figure 4a shows the FT-IR spectra of the obtained oleogels while Figure 4b presents the FT-IR spectrum of lidocaine.

**Figure 5:**
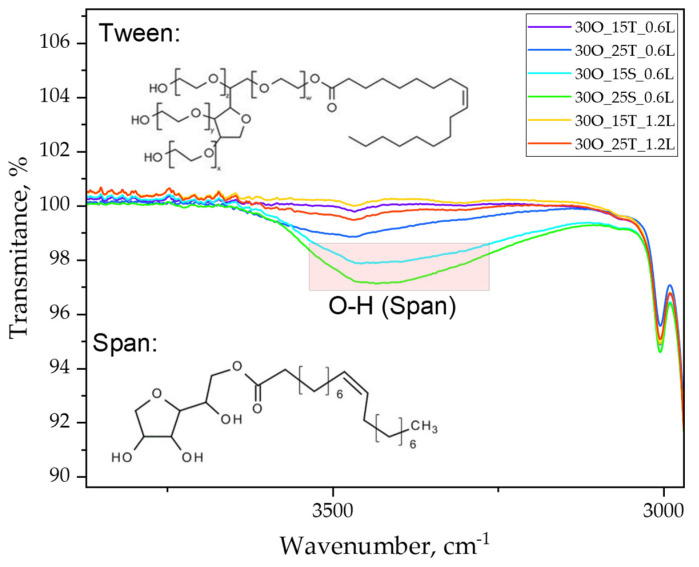
Approximation of the FT-IR spectrum from Figure 4a after overlaying all spectra, revealing the precise depth of absorption bands originating from the surfactants TWEEN 80 and SPAN 80.

The above graphs present a comparison of FT-IR spectra for all studied samples—both immediately after synthesis and the spectrum for pure lidocaine for the purpose of identifying characteristic groups. Table 1 provides the relevant compilation for the analysis of the obtained spectra. Figure 5 presents a zoomed-in view, highlighting the changes in the intensity of bands for hydroxyl groups present in the structure of the emulsifiers used.

This study confirmed the presence of characteristic groups derived from the substrates used in the obtained materials and allowed for the observation of differences in band intensity depending on the amount of substrate applied during the synthesis process.

### 2.3. Sorption Properties of Oleogels

**Figure 6:**
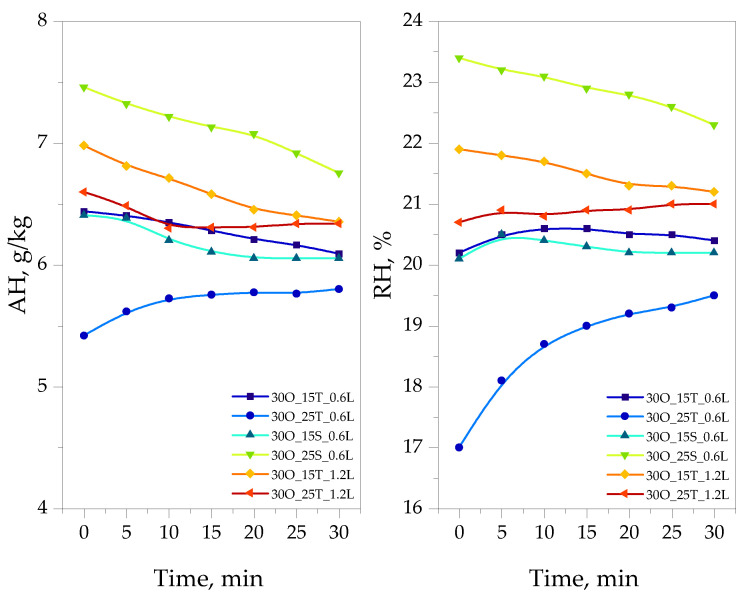
Changes in the absolute (**AH**) and relative (**RH**) humidity of oleogels over time.

The results of the performed experiments aimed at determining the sorption ability of oleogels are presented in Figure 6.

The study of the material’s reaction to moisture present in the environment allows for the assessment of the material’s interaction with the environment, which may simulate its potential applications. A detailed discussion of the obtained results is presented in the following subsection.

### 2.4. Wettability of Oleogels

**Figure 7:**
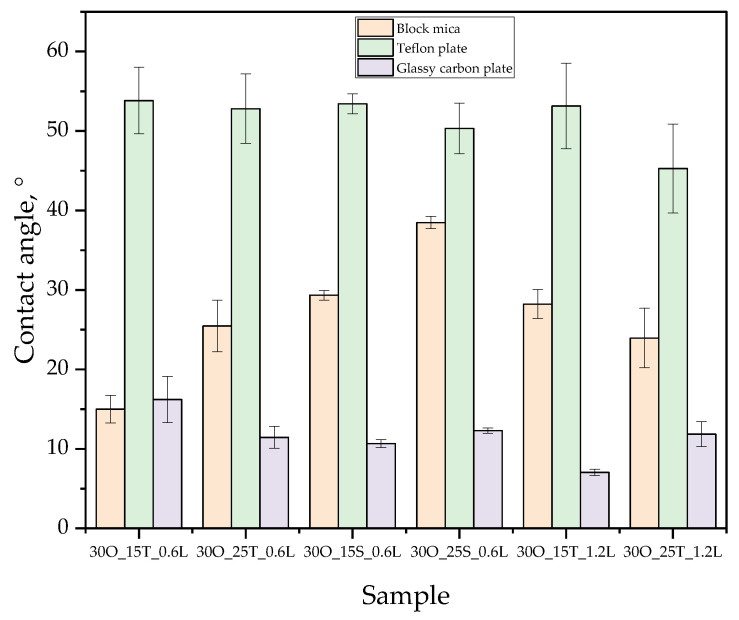
Contact angles for the obtained oleogel materials with standard deviations calculated from five repetitions.

**Figure 8:**
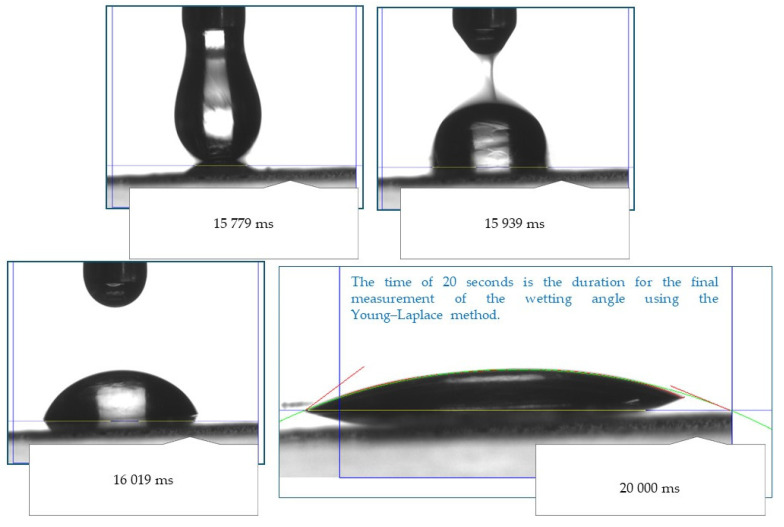
Images of the oleogel drop behavior during the initial contact with the test material.

Figure 7 compiles wetting angles for oleogels determined for three types of materials, i.e., block mica, Teflon plate, and glassy carbon plate. In Figure 8, the images showing the behavior of a drop of oleogel after initial contact with the test material are presented.

The study of surface wettability allows for the characterization of the hydrophilic or hydrophobic nature of the obtained materials. The diagram presented in Figure 8 illustrates the measurement principle applied during all measurements conducted for the obtained materials. Collecting measurements after a specified time for the tested materials is essential and enables the acquisition of more reliable research results.

### 2.5. Results of Studies on Rheological Properties of Oleogels

**Figure 9:**
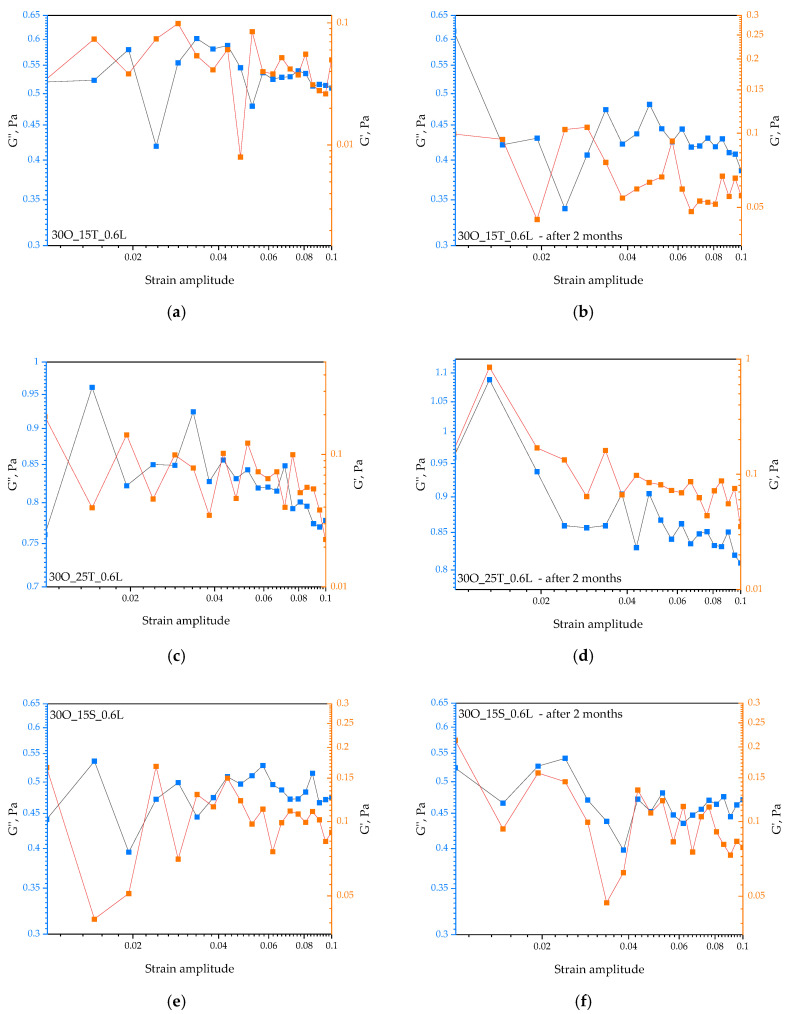

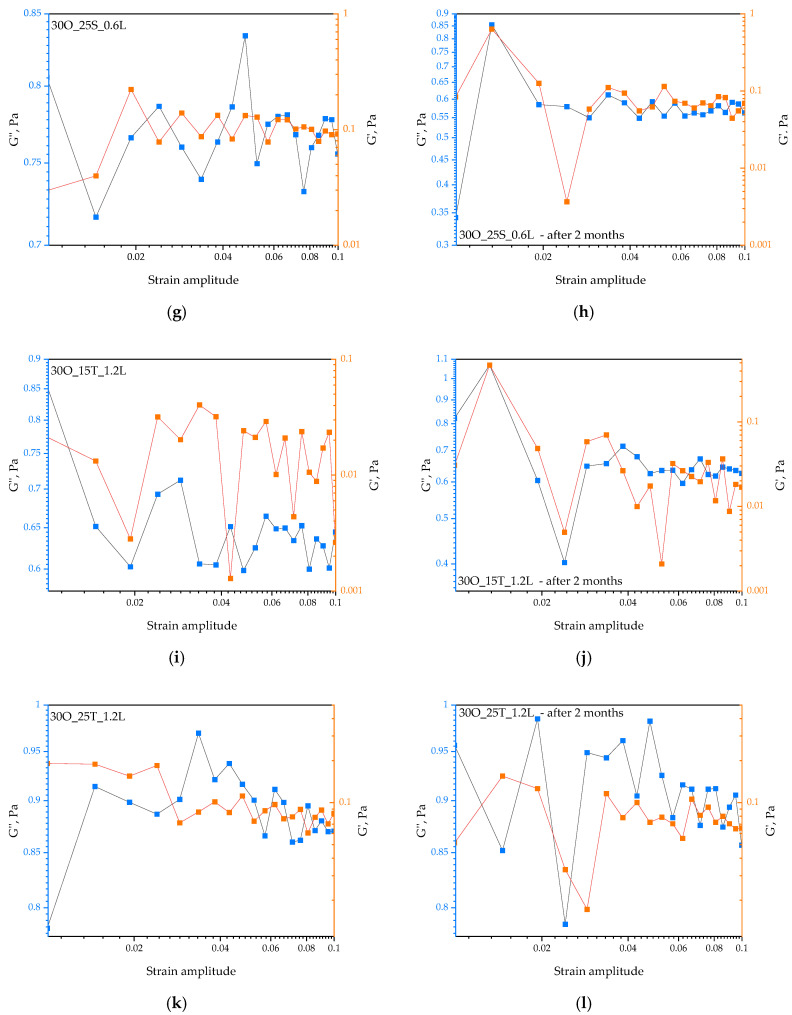
Summary of G′ and G″ for materials tested immediately after synthesis (**a**,**c**,**e**,**g**,**i**,**k**) and 2 months after synthesis (**b**,**d**,**f**,**h**,**j**,**l**).

In Figure 9, the results of the performed rheological analyses are presented.

## 3. Discussion

Based on the results obtained from the MultiScan analysis, we can assess the stability of the emulsion after two months of storage at 6 °C and attempt to interpret the type of emulsion present in our materials. Analyzing Figure 2, we can begin by comparing the transmission spectrum for sample 30O_15T_0.6L and the same sample stored for two months (30O_15T_0.6L (old)). It is evident that the oleogel examined immediately after synthesis shows a significantly different spectrum compared to the material stored for two months, which may indicate changes occurring in the material over time.

Focusing on the detailed interpretation of the spectrum for the 30O_15T_0.6L sample, we observe that up to the 4 mm position, the light does not pass through the sample. This is most likely due to significant scattering, as nothing suggests that absorption would occur here, considering the nature of the components in the sample. Next, we observe an increase in transmission to almost maximum levels, indicating that the material is almost fully transparent to light in this range.

However, the 30O_15T_0.6L (old) sample exhibits a different behavior. From the start, we observe a transmission increase to 72% compared to the freshly synthesized sample. It is likely that prolonged storage caused the partial separation of the emulsion, leading to reduced light scattering and thus higher transmission relative to the initial sample. A subsequent drop in transmission can be observed in the spectrum, which may suggest the formation of heterogeneities or aggregates as a result of prolonged storage. Following this, transmission levels rise again, but only to around 72%, compared to about 97% in the “fresh” sample. This may suggest the continued presence of an emulsion, albeit with an altered structure, indicating partial destabilization caused by the storage process.

Comparing the MultiScan results with the photograph presented in Figure 1, we can initiate an interesting discussion. Figure 1 shows the appearance of the systems immediately after synthesis and after two months of storage. In the photograph, we can observe the separation of phases in the sample with the naked eye. For the phase comparison between 30O_15T_0.6L and 30O_15T_0.6L (old), we see that the phase separation visible in the photo is also reflected in the transmission spectrum. At 25 mm, after 30 min, we observe a slight increase in transmission, which most likely corresponds to the phase separation seen in the photograph. The increase in transmission suggests that above the visible phase boundary, we are most likely observing just oil, which has a lower capacity for light scattering, thereby increasing transmission. This is, of course, an undesirable phenomenon as it indicates the destabilization of the final product.

The destabilization is observed in all samples except those obtained using SPAN as the emulsifier. Based on this, we can conclude that lidocaine does not affect the stability of the emulsion, but the choice of emulsifier does. Additionally, a long storage period allows for the creation of a stable emulsion throughout that time, provided that the appropriate amount of emulsifier is used. The samples marked with a pink circle in Figure 1 show no visible phase separation, even after two months of storage. However, when analyzing the transmission graph for these materials, we see that the sample containing 15 g SPAN exhibited changes in transmission after two months, indicating that only the sample containing 25 g SPAN remained stable for the entire two-month period.

The overall conclusions from this study are that lidocaine does not influence emulsion stability; rather, it is the emulsifier that plays a key role. The difference between emulsions stabilized with SPAN and those with TWEEN is due to the fact that TWEEN is a hydrophilic surfactant (with a high HLB, around 15), meaning it stabilizes oil-in-water (o/w) emulsions, where the oil phase is dispersed in the water phase. TWEEN 80 prefers interactions with water and stabilizes emulsions with a more “aqueous” structure. SPAN, on the other hand, is a lipophilic surfactant (with a low HLB, around 4.3), which is better at stabilizing water-in-oil (w/o) emulsions, where the water phase is dispersed in the oil phase. SPAN 80 prefers interactions with oil and stabilizes emulsions with a more “oily” structure. The role of this surfactant in stabilizing w/o emulsions has also been described in [32,33].

To investigate the structural differences between the emulsifiers used and to assess their impact on the material’s structure, FT-IR analysis was conducted, as presented in Figure 4 and Figure 5. Figure 4a shows the spectrum comparison for all samples, while Figure 4b presents the spectrum for lidocaine alone. Analyzing the full set of spectra, we can observe bands corresponding to C-H (alkanes) and C=O (esters), which originate from components such as olive oil, TWEEN, and SPAN. Due to its structure, TWEEN contains more ester groups compared to SPAN, and this can be observed in the reduced intensity of the C-O-C band, marked in Figure 4a for samples containing SPAN, i.e., 30O_15S_0.6L and 30O_25S_0.6L.

Furthermore, the spectra show C-N groups originating from lidocaine, but we do not observe a significant deepening of this band depending on the amount of lidocaine in the sample. This is likely due to the limitations in the accuracy of this particular method. In addition, Figure 5 provides a zoomed-in view of the 4000 to 3000 cm^−1^ range from Figure 4a. The aim was to examine the intensity of bands originating from the emulsifiers and to check for any potential differences in this region (since spectral separation often leads to the flattening of the spectra, making it difficult to observe differences, we decided to isolate the section that exhibited the most prominent differences).

In Figure 5, we can see that the bands from SPAN are significantly more intense than those from TWEEN. Moreover, a higher quantity of SPAN in the sample further confirms this relationship, as the band for the sample with 25 g of SPAN is more intense than the one containing 15 g of SPAN. In TWEEN 80, many hydroxyl groups may be more “hidden” within the structure, especially when interacting with other components such as olive oil or lidocaine. These groups may be less capable of forming strong hydrogen bonds, which could result in a lower intensity of the O-H band. Additionally, the structure of SPAN 80 is less “congested” on the hydrophilic side, which may increase the availability of O-H groups for detection.

Staying within the scope of comparing the properties of emulsifiers and the impact of hydroxyl groups in their structures on material characteristics, a moisture measurement study was conducted. The samples were sealed in an airtight container with temperature monitoring. As shown in the results presented in Figure 6, the sample containing 15 g of TWEEN and 0.6 g of lidocaine quickly reached moisture equilibrium—within just 5 min. This indicates that this system responded rapidly to environmental conditions and stabilized in a very short time.

The next sample, which contained 25 g of TWEEN and the same amount of lidocaine, released much more moisture into the environment throughout the experiment. Interestingly, in the case of the sample with 25 g of TWEEN but 1.2 g of lidocaine, this effect was not observed, likely because the higher lidocaine content blocked this effect and allowed the material to reach moisture equilibrium.

The sample containing less SPAN and lidocaine behaved similarly to the sample with less TWEEN, suggesting that 15 g of SPAN provides a similar ability to retain moisture as 15 g of TWEEN, although through different mechanisms of water interaction. For the sample with a higher SPAN content, the decrease in humidity over time indicates that the sample started absorbing moisture from the environment, lowering the RH level. This may confirm the more intense hydroxyl group band observed in the FT-IR spectrum, where a greater number of hydroxyl groups enhances their ability to interact with moisture, resulting in the observed decrease in RH levels.

Next, a surface wettability study was conducted on different materials, and the results are presented in Figure 7. The general principle for interpreting this analysis is that a small contact angle indicates good surface wettability, i.e., better adhesion of the sample to the surface. In contrast, a larger contact angle suggests weaker interactions with the surface [34].

Analyzing the results for the mica surface, we observe that samples containing SPAN exhibit larger contact angles, which is attributed to the hydrophobic nature of this emulsifier [35]. Consequently, the larger the amount of SPAN (e.g., 25 g), the greater the contact angle, compared to the sample containing 15 g of SPAN. Mica, being a hydrophilic material [36], highlights how the addition of a hydrophobic substance in the samples decreases surface wettability.

Regarding the effect of lidocaine on surface wettability, when there is a smaller amount of TWEEN in the sample, we notice reduced wettability, as evidenced by the larger contact angle. However, in the sample with a higher TWEEN concentration, the increased amount of lidocaine does not significantly impact the contact angle. This suggests that an appropriate ratio of emulsifier to lidocaine can result in a product that maintains consistent wettability, independent of minor variations in lidocaine content.

Focusing on the results for the Teflon surface, we observe no significant differences between the samples. The results are quite similar, and no clear dependencies emerge. This is due to the hydrophobic nature of both the oleogels and the Teflon surface itself [37], which limits the ability to draw meaningful conclusions, as the differences in contact angle values fall within the range of the standard deviations.

The final surface tested was glassy carbon, which has relatively balanced properties—neither distinctly hydrophobic nor hydrophilic [38]. Based on the results, we can observe that samples containing SPAN show the most consistent results. This consistency also appears in the mica test, which is important as it suggests that these oleogels likely have the most homogeneous structure and the most stable emulsion. This conclusion was also confirmed through MultiScan tests and by examining photographs taken with a highly sensitive camera.

The final study was a rheological analysis, where materials were tested immediately after synthesis and also after two months of storage (Figure 9). The rheological study also confirmed the results from previous analyses, especially those obtained using the MultiScanning technique. Based on the results presented in Figure S1a, we can observe that the sample with a higher amount of SPAN and 0.6 g of lidocaine exhibits a modulus with slight fluctuations, suggesting that the material becomes more “solid-like” and well maintains its structure under stress. Consequently, we can conclude that this sample immediately after synthesis has the most stable and increasing elastic modulus, indicating that it is a material with a strong structure, resistant to deformation. Regarding the impact of storing materials for 2 months, we can confirm that prolonged storage at 6 °C has positively affected the structural stability of most samples, improving their mechanical properties, except for Sample 30O_15T_1.2L, which still shows signs of instability.

Analyzing the graphs presented in Figure S2, we can observe the absence of intersection points (G′ = G″) for the samples tested immediately after synthesis, suggesting that all samples after synthesis have dominant viscous properties. The energy loss associated with viscous deformation (G″) is greater than the energy recovered by the material during elastic deformations (G′). The most balanced viscoelastic properties (the smallest differences between G′ and G″) are exhibited by samples 30O_25S_0.6L and 30O_25T_1.2L. Although there are no clear intersection points, the differences between G′ and G″ are smaller, indicating the greater elasticity of these samples compared to others. Intersection points (G′ = G″) usually indicate a change in the dominant properties of the material—where elasticity dominates, G′ > G″, and where viscosity dominates, G″ > G′. The absence of an intersection point suggests that the material consistently maintains the dominance of one property over the other.

If we focus on comparing these properties for materials tested after the 2-month storage period at 6 °C, we can observe a lack of intersection points in each of the samples. Sample 30O_25S_0.6L shows a favorable change in terms of elasticity after 2 months, suggesting that it may be more suitable for applications requiring better mechanical properties.

## 4. Materials and Methods

### 4.1. Materials

The main components of the oleogel were highly refined olive oil (low acidity), TWEEN 80, SPAN 80, and lidocaine. All materials were sourced from Sigma Aldrich (Saint Louis, MO, USA). TWEEN 80 (Polysorbate 80) is an amphiphilic surfactant that reduces surface tension, aiding in the formation of stable emulsions, while SPAN 80 (sorbitan monooleate) is a lipophilic emulsifier effective in stabilizing water-in-oil emulsions. Lidocaine, a neuroprotective agent with a purity of ≥98% (GC), was also included in powder form. During the synthesis and all investigations a double deionized distilled water was applied. All reagents were used as received without further purification.

### 4.2. Synthesis of Olive Oil-Based Oleogels Containing Lidocaine

The first step involved dissolving the appropriate amount of lidocaine in olive oil at 60 °C, while stirring for 30 min (8500 rpm) using a Unidrive X1000 CAT (Ingenieurbüro CAT, M. Zipperer GmbH, Ballrechten-Dottingen, Germany) homogenizer to obtain a homogeneous mixture. In the second stage, the appropriate amount of emulsifier was added to the mixture (still at 60 °C) and vigorously stirred using the same homogenizer (20,000 rpm). The material was then transferred into sterile glass cells, which were placed at 6 °C for 48 h. Afterward, the studies described in this article were conducted. The tested series was divided into several vials, with part of each series stored for 2 months at 6 °C before being subjected to the tests.

In Table 2, the compositions of all prepared samples are compiled.

After synthesis, the obtained samples were subjected to numerous investigations aimed at characterizing their stability, chemical structure, sorption, and rheological properties as well as wettability.

### 4.3. Verification of Stability of Obtained Oleogels

The stability of oleogels was measured via a stability MultiScan MS20 system. The first step of the research included measuring samples immediately after synthesis. Next, the oleogels were stored at 6 °C for two months and investigated again. Measurements were performed at room temperature.

### 4.4. Characterization of Oleogels Using FT-IR ATR Spectroscopy

The presence of individual functional groups within the structure of developed materials was verified via Fourier-transform infrared (FT-IR) spectroscopy. The study was conducted using a Thermo Scientific Nicolett iS5 (Thermo Fisher Scientific Inc., Waltham, MA, USA) spectrometer equipped with an attenuated total reflection (ATR) attachment containing a diamond crystal. The measurement range was 4000–500 cm^−1^ (32 scans at 0.4 cm^−1^ resolution). Analysis was conducted at room temperature.

### 4.5. Sorption Ability of Developed Systems

In order to characterize the sorption properties of the obtained oleogels, experiments were conducted involving determining the relative humidity (RH) changes within the tightly closed Duran glass bottle containing two Petri dishes: one with a tested sample (weighing 1.0 g) and a second one with a double-deionized distilled water. The experiments were conducted for 30 min while the relative humidity was read every 5 min. In addition, the temperature changes in the test vessel were also monitored.

The determined relative humidity values allowed the researchers to calculate absolute humidity (AH) using the following equation (Equation (1)):

(1) $$AH=\left[\left(\frac{1320.65}{T+273.15}\right)\cdot 10\left(\frac{7.4475\cdot T}{T+233.71}\right)\right]\cdot RH$$

where:

- AH is absolute humidity, g/kg;
- RH is relative humidity, %.

Measurements were carried out using a thermo/hydrometer Elmetron PWT-401 (Elmetron GP, Zabrze, Poland) which enables simultaneous measurements of temperature and relative humidity (range 0–100%; resolution 0.1% RH).

### 4.6. Analysis of Wetting Properties of Oleogels

The wetting properties of the obtained oleogel samples—directly after synthesis and after two months of the storage—were determined using the Drop Shape Analyzer Kruss DSA100M optical contact angle measuring instrument (A. KRÜSS Optronic GmbH, Hamburg, Germany) equipped with a digital camera (200 fps; the device applies a digital image processing algorithm to determine the contact angles by means of the Laplace–Young approximations or tangents) and an optical microscope. Every measurement was performed using sterile syringe needles (NE 44, Kruss Gmbh, Hamburg, Germany). The oleogel samples were dispensed onto the test substrate (three types of substrates were analyzed, i.e., block mica, Teflon plate, and glassy carbon plate) using a syringe positioned inside the environmental chamber. The needle’s position was adjusted accordingly. As each sample spread across the surface, forming a layer, it was necessary to wait 10 s for the sample to stabilize. Once the oleogel had settled, the contact angles were recorded on video and measured after setting the baseline. After the liquid on each substrate stabilized, the device displayed the calculated contact angle values. Such analysis was performed in triplicates for each sample. The procedure applied was analogous to the procedure described in detail in our previous paper [39].

### 4.7. Studies on Rheological Properties

A significant aspect of the research was to verify the rheological properties of the obtained oleogels as well as the potential change in these properties after two months of storage. For this purpose, the Malvern Bohlin Gemini II (Malvern Panalytical Ltd., Cambridge, UK) rheometer, additionally equipped with sample temperature monitoring via a Peltier system and the software Bohlin R6.51.0.3 (Malvern Panalytical Ltd., Cambridge, UK), was employed. During the experiments, 1.0 g of each sample was used, while the gap between the plate probe (angle = 4°; diameter: 40 mm) and the cone was set to 1 mm. The complex shear modulus (denoted G* = G′ + iG″) was determined as a function of increasing applied strain amplitude at a fixed frequency (equal to 1 Hz) and received based on the amplitudes and phases of the first harmonic strain signals. A frequency sweep test (within the range 0–10 Hz) was utilized to obtain the elastic modulus (G′) and loss modulus (G″) of the test samples in the linear viscoelastic region (LVR). All measurements were applied at 25 ± 1 °C.

## 5. Conclusions

Based on the conducted studies on emulsions containing lidocaine, the following conclusions can be drawn regarding their potential application in medicine. Emulsions stabilized with SPAN showed better stability and lower tendencies for phase separation compared to those stabilized with TWEEN. The proper choice of emulsifier is crucial for obtaining stable oleogels, which is important in terms of their storage and clinical application. Lidocaine does not significantly affect the stability of the emulsions, suggesting that its presence can be well tolerated in formulations without disrupting the physicochemical properties of the final product. Samples containing higher amounts of SPAN exhibited moisture absorption capacity, which may be relevant for their use in therapies where moisture control and moisturizing properties are essential. Oleogels with lidocaine stabilized with SPAN demonstrated favorable mechanical properties, which could translate to better application and adherence to the skin in therapeutic uses. Due to their stability, rheological properties, and ability to retain moisture, these emulsions have significant potential in medicine, particularly in pain relief and dermatological formulations where long-lasting effects and application comfort are important.

In summary, oleogels with lidocaine, especially those stabilized with SPAN, may be promising candidates for medical applications.

## Figures and Tables

**Table 1:** Summary of all vibrations with their corresponding chemical compounds involved in the obtained materials.

Wavenumber [cm^−1^]	Type of Vibration	Assigned Group	Component
3400–3200	Stretching	O-H (hydroxyl)	SPAN, TWEEN
2950–2850	Symmetric and asymmetric stretching	C-H (alkanes)	Olive oil, TWEEN 80, SPAN 80
1740–1735	Stretching	C=O (ester)	Olive oil, TWEEN 80, SPAN 80
1650–1620	Stretching	C=O (amide)	Lidocaine
1200–1100	Stretching	C-O-C (ether)	TWEEN 80, SPAN 80
1200–1350	Stretching	C-N (amine)	Lidocaine
1480	Bending	C-H (aromatic groups)	Lidocaine
758	Bending	C-H (aromatic groups)	Lidocaine

**Table 2:** Compositions of prepared oleogels.

Sample Name	Olive Oil, g	TWEEN 80, g	SPAN 80, g	Lidocaine, g
30O_15T_0.6L	30	15	-	0.6
30O_25T_0.6L		25	-	
30O_15S_0.6L		-	15	
30O_25S_0.6L		-	25	
30O_15T_1.2L		15	-	1.2
30O_25T_1.2L		25	-	

## Data Availability

The original contributions presented in the study are included in the article and Supplementary Materials, further inquiries can be directed to the corresponding authors.

## Supplementary Materials

The following supporting information can be downloaded at: https://www.mdpi.com/article/10.3390/ijms252011067/s1.
