# Supplementary material for: Study on the Effect of Emulsifiers on the Properties of Oleogels Based on Olive Oil Containing Lidocaine
Source: Int J Mol Sci. 2024 Oct 15;25(20):11067. doi: 10.3390/ijms252011067 (PMC11508005; doi:10.3390/ijms252011067)
Supplement: Supplementary file 1 [file ijms-25-11067-s001.zip › ijms-3254909-supplementary.pdf]

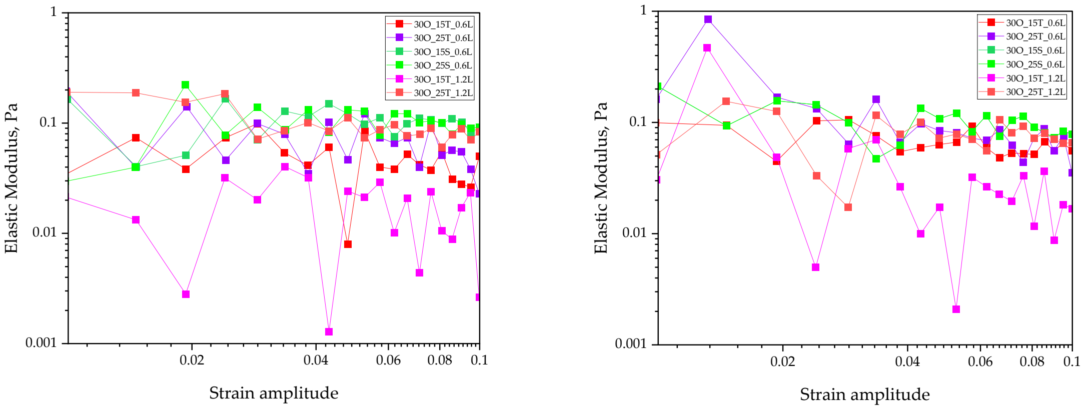

(**a**) (**b**)

Figure S1. Dependance between the elastic modulus and the strain amplitude of oleogels measured directly after their synthesis (a) and after two months (b).

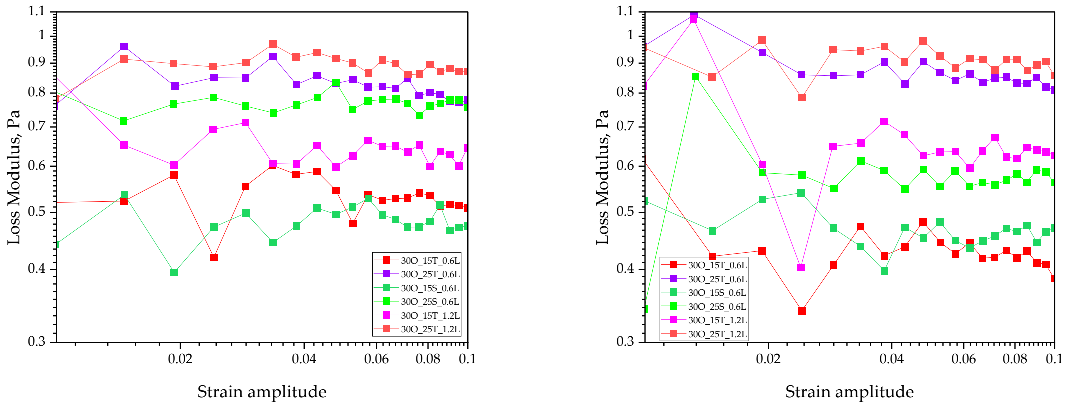

(**a**) (**b**)

Figure S2. Dependance between the loss modulus and the strain amplitude of oleogels measured directly after their synthesis (a) and after two months (b).
